# Die Macht der Psychiatrie: Reflexionen zu historischer Aufarbeitung und Aktualität

**DOI:** 10.1007/s00048-025-00427-3

**Published:** 2025-09-19

**Authors:** Paulina S. Gennermann

**Affiliations:** Fachbereich Geschichte und Kulturwissenschaften, Neueste Geschichte, Philipps-Universität Marburg, Wilhelm-Röpke-Str. 6c, 35032 Marburg, Deutschland

Borck, Cornelius und Gabriele Lingelbach (Hg.) 2023. *Zwischen Beharrung, Kritik und Reform. Psychiatrische Anstalten und Heime für Menschen mit Behinderung in der deutschen Nachkriegsgeschichte*. Frankfurt am Main/New York: Campus, brosch., 429 S., 48,00 €, ISBN: 9783593516585.

Shorter, Edward 2021. *The Rise and Fall of the Age of Psychopharmacology*. Oxford: Oxford University Press, brosch., 448 S., 62,67 €, ISBN: 9780197574461.

Söhner, Felicitas 2019. *Psychiatrie-Enquete: mit Zeitzeugen verstehen. Eine Oral History der Psychiatriereform in der BRD*, herausgegeben von Thomas Becker und Heiner Fangerau. Köln: Psychiatrie-Verlag, brosch., 208 S., 25,00 €, ISBN: 978-3-88414-953‑9.

Am 12. Februar 2025 berichtete *Tagesschau Online* über den deutschen Kinder- und Jugendpsychiater Michael Winterhoff. Der einst „gefragt[e] Gast in Talkshows, Podiumsdiskussionen und Vortragsreihen“ und „wohl bekannteste Kinderpsychiater Deutschlands“ musste sich wegen des Tatverdachts der gefährlichen Körperverletzung vor Gericht verantworten. Winterhoff hatte insbesondere Heimkindern Neuroleptika verordnet, zu denen bisweilen nicht ausreichend Studien zur Wirkung bei Kindern vorlagen. Es traten Nebenwirkungen auf, die scheinbar nicht ernst genommen oder durch die Gabe weiterer Medikamente gedämpft wurden (Rosenbach 2025). Dieses Agieren des renommierten Kinderpsychiaters verdeutlicht, wie medizinische Autoritäten Handlungsspielräume nutzen und wie angreifbar vulnerable soziale Gruppen dadurch sein können. Die Psychiatrie – womit hier sowohl die Institution als auch ihre Angehörigen, ihre Infrastruktur und ihr Netzwerk gemeint ist – besitzt unterschiedliche Formen von Wirkmacht und Einfluss. Während der Fall Winterhoff vor allem die gesellschaftliche Wirkmacht der Psychiatrie demonstriert, veranschaulicht ein nicht weniger aufsehenerregender Pressebericht aus den USA ihre politische Wirkungsmacht. Am 31. März 2025 veröffentlichte die *TAZ Online* einen Kommentar über republikanische Politiker:innen aus Minnesota, die eine Einstufung von Kritik an Donald Trump als psychische Erkrankung diskutierten. Auch wenn es als unwahrscheinlich gilt, dass ihr Vorstoß zur Anerkennung des „Trump Derangement Syndrome“ (TDS) erfolgreich ist, macht das Vorhaben auf den potenziellen politischen Einfluss der Psychiatrie als Institution und der psychiatrischen Diagnostik als Methode aufmerksam und deutet ihre mögliche politische Instrumentalisierung an (Schöpfer 2025). Regimekritik wird in die psychiatrische Diagnostik integriert und so eine Regierung gegen potenzielle Angriffe verteidigt. Die Pathologisierung der Kritik dient einerseits der sozialen und andererseits der räumlichen Isolierung – insofern, als die Psychiatrie zum Verwahrungsort für Betroffene werden kann.

Sowohl der Fall Winterhoff als auch die Diskussion um TDS bezeugen die gesellschaftliche und politische Macht der Psychiatrie und der psychiatrischen Diagnose bei Normsetzung und -durchsetzung hinsichtlich individueller Denk- und Handlungsweisen. An unterschiedlichen Stellen entstehen Handlungsspielräume für die Psychiatrie selbst, gleichzeitig kann sie von anderen Akteur:innen instrumentalisiert werden. In der aktuellen Medizin- und Psychiatriegeschichte haben Studien zu Einfluss und Macht der Psychiatrie einen hohen Stellenwert und ergänzen damit die Forschungen zur Funktion psychiatrischer Einrichtungen und zum Umgang mit psychisch Erkrankten (Hähner-Rombach & Nolte 2017; Schädle-Deininger 2021; Quensel 2018). Auch im (post-)kolonialen und globalen Kontext haben sich Wissenschaftler:innen mit der Entwicklung der psychiatrischen Forschung und ihren medizinischen als auch sozialen Auswirkungen auseinandergesetzt (Chi 2024; Kirmayer 2020; Müller 2017; Muños 2018). Die Sensibilität für den Umgang mit vulnerablen Gruppen, für die Globalität des Phänomens Psychiatrie und für Kritik an der bis dato gängigen glorifizierenden Psychiatriegeschichtsschreibung nimmt zu. Dies wird auch in den Publikationen von Cornelius Borck und Gabriele Lingelbach, Edward Shorter und Felicitas Söhner deutlich. Sie heben die gesellschaftliche und politische Wirkmacht der Psychiatrie und des psychiatrischen Apparats insgesamt hervor, führen die Stimmen betroffener Personen zusammen und öffnen in diesem Zusammenspiel eine innovative und vielschichtige Perspektive auf Psychiatrie, Psychiatriegeschichte und die Geschichte der Psychopharmakologie. Ein gemeinsamer Schwerpunkt liegt dabei auf der kritischen Reflexion der Historiographie als Erfolgsgeschichte.

## Neue Perspektiven auf Psychiatrie und Psychopharmakologie

Der Sammelband *Zwischen Beharrung, Kritik und Reform. Psychiatrische Anstalten und Heime für Menschen mit Behinderung in der deutschen Nachkriegsgeschichte* von Cornelius Borck und Gabriele Lingelbach verbindet erfolgreich Psychiatriegeschichte und Disability History und unterstreicht die Wichtigkeit interdisziplinärer Herangehensweisen in diesen Forschungsfeldern. In vier Abschnitten werden verschiedene Blickwinkel und Entwicklungen psychiatrischer Anstalten sowie Einrichtungen für Menschen mit Behinderungen untersucht und miteinander in Beziehung gesetzt. Der Schwerpunkt liegt auf der jungen Bundesrepublik Deutschland der Nachkriegszeit bis in die 1990er Jahre.

Im ersten Teil wird die institutionelle Rahmung in ihren Alltagspraktiken untersucht. Es wird deutlich, dass der Anstaltsalltag häufig nicht den Anforderungen seitens Politik, Medizin und Öffentlichkeit entsprach. Dies lag unter anderem, so Thomas Beddies, an den zahlreichen finanziellen, personellen und infrastrukturellen Mängeln, die die Nachkriegszeit prägten. Des Weiteren werden die Verbindungen gesellschaftlicher Lebensweisen und Normen mit der psychiatrischen Rahmung herausgearbeitet. Insbesondere am Beispiel der Arbeitsfähigkeit zeigen Uwe Kaminsky und Franz-Werner Kersting die Verschränkung von Therapieformen und sozialen Werten. Ausgehend davon greift dieser Teil auch die psychiatrischen Bemühungen um die eigene Verwissenschaftlichung auf. Anstalten sollten als vollwertige Fachkrankenhäuser definiert und psychiatrisches Wissen als methodisch zertifiziert und therapeutisch nützlich anerkannt werden. Dies lag quer zur Etablierung der „Kennerschaft“ vonseiten der Psychiater:innen, so Stefanie Coché. Das geschulte Auge des Psychiaters sollte auch ohne naturwissenschaftliche Standards und Vorgaben Erkrankungen erkennen können. Dadurch erhielten Psychiater:innen als Personen eine mächtige Position; ihre „Kennerschaft fungierte als Inklusions- und Exklusionsmittel und zur Identifizierung ‚des Fremden‘ in der Gemeinschaft“ (Coché 2023: 107).

Im zweiten Teil werden Bemühungen um bessere Behandlungen und soziale Gerechtigkeit in den Blick genommen. Die Psychiatrie-Enquete von 1975 gilt dabei gemeinhin als einschneidendes Ereignis in der Psychiatriegeschichte Westdeutschlands. Sie rückte die bestehenden Probleme und Missstände der psychiatrischen Versorgung ins Licht der Öffentlichkeit und forderte umfassende Reformen (Löffelbein 2023: 223). Die Realität sah jedoch bis in die 1990er Jahre anders aus. Zwar wurde der Umgang mit psychisch kranken Menschen vor allem hinsichtlich der Zwangseinweisung problematisiert und im Hinblick auf das Grundgesetz und vor dem Hintergrund der nationalsozialistischen Vergangenheit neu beurteilt, wobei sowohl die Wertigkeit als auch die Rechte der Betroffenen diskutiert wurden, so Frank Sparing. Allerdings blieb der Charakter der Einrichtungen als Verwahranstalt erhalten. Ebenso hielt sich der stigmatisierende Charakter der Diagnostik: Für eine Vielzahl an Menschen – Erwachsene wie Kinder – bedeutete eine psychiatrische Diagnose eine langfristige gesellschaftliche Stigmatisierung. Dies betraf sowohl psychisch kranke Menschen als auch Menschen mit Behinderungen, wie Christine Hartig herausarbeitet.

Der dritte Teil gibt den Betroffenen selbst eine Stimme in der Geschichtsschreibung. Herangezogen werden von Burkhart Brückner dafür persönliche Dokumente und Kunstwerke wie Zeichnungen, von Monika Ankele Studien zur Bedürfnisanalyse psychiatrischer Patient:innen und von Christof Beyer Patient:innenzeitungen. Die Betroffenenperspektive verdeutlicht, dass die Einrichtungen nicht nur als medizinische Institutionen zu verstehen sind, sondern auch als Wohn- und Lebensraum (Ankele). Die Anforderungen an diese Räume und ihre Wahrnehmung variierten je nach Perspektive. Um mit der Außenwelt in Kontakt zu treten, gründeten Patient:innen Verbände und Zeitungen. Sie wollten sich Gehör verschaffen und auf etwaige Missstände aufmerksam machen (Brückner; Beyer). Dies wurde allerdings unter anderem von Mitarbeitenden als „Störung des Betriebs“ (Beyer 2023: 324) wahrgenommen, während Betroffene selbst Diskriminierung erlebten.

Der abschließende vierte Teil greift die kritische Betrachtung psychiatrischer Maßnahmen und ihrer gesellschaftlichen Regulierung hinsichtlich medizinischer und pharmakologischer Praktiken auf. Der Fokus liegt auf dem Umgang mit Evidenzbasierung in der Psychiatrie und dem Einsatz von Psychopharmaka. Da psychopharmazeutische Stoffe am Menschen eingesetzt und ausprobiert wurden, spricht einiges dafür, diese Praktiken unter dem Aspekt der Menschenexperimente zu diskutieren, so Jonathan Holst. Viola Balz hinterfragt die Menge verwendeter Psychopharmaka, wobei nicht nur der „ärztlich verordnete Massenkonsum“ (Balz 2023: 352) untersucht wird, sondern auch ihr Einsatz als Disziplinierungsmaßnahme. Die Untersuchung des Umgangs mit Psychopharmaka als „Signum eines psychiatrischen Unrechtssystems“ (Holst 2023: 383) sowie dessen zeitgenössischer Rezeption ergänzt dabei gut die Perspektiven Betroffener, die weniger die Psychiatrie und die Medikamente an sich, sondern die herrschenden Zustände kritisierten.

Die Monographie *Psychiatrie-Enquete: mit Zeitzeugen verstehen. Eine Oral History der Psychiatriereform in der BRD* von Felicitas Söhner eröffnet mittels zahlreicher Zeitzeug:inneninterviews eine wichtige Einsicht in die damalige Wahrnehmung von Einfluss und Wirkung der Psychiatrie-Enquete von 1975. Söhner diskutiert die äußeren Rahmenbedingungen der Enquete, die Vernetzung und Weiterbildung beteiligter Akteur:innen sowie die internen Entwicklungen im psychiatrischen Milieu und bringt dafür ein Generationenkonzept in Anschlag. Das Werk interferiert mit dem Sammelband von Borck und Lingelbach insofern, als Alltagspraktiken und Veränderungen im Psychiatriealltag diskutiert und die Analyse der Veränderungsbewegungen anhand intimer Einblicke in die Entstehung der Psychiatrie-Enquete fortgeführt werden.

Die Resonanz von Reformideen entstammte, so Söhner, insbesondere dem internationalen fachlichen Austausch. Aus Großbritannien und den USA, aber auch aus Frankreich und Italien erreichte die Bundesrepublik Deutschland Kritik am vorherrschenden psychiatrischen Krankenhaussystem. Vor allem sozialpsychiatrische Strömungen fanden ihren Weg in die Bundesrepublik. Akteur:innen wurden in diesem Zusammenhang zunehmend für die Missstände in der Psychiatrie sensibilisiert. Dabei stachen vor allem die sogenannte „Zwischengeneration“ und die „kritischen Jungen“ hervor, die sich zwar durch eine bestimmte Richtung des Reformwillens auszeichneten, sich in ihren Ansichten und Herangehensweisen allerdings nicht selten stark unterschieden. Während sich Erstere durch wissenschaftliche Karrieren nach 1945 und einer daran angeschlossenen Reformbereitschaft auszeichnete, waren Letztere insbesondere durch die Achtundsechzigerbewegung geprägt und empfanden eine dringende Notwendigkeit, das bestehende autoritäre Regime der Psychiatrie zu verändern. Damit zusammenhängend stellt Söhner heraus, dass es unterschiedliche Reformansätze gab, die sich in lokalen und regionalen Netzwerken jeweils spezifisch ausbildeten und verbreiteten. So fand sich beispielsweise im Rhein-Main-Kreis vor allem die „Zwischengeneration“ zusammen, im Mannheimer Kreis die „kritischen Jungen“. In diesem Zusammenhang wurde der transdisziplinäre Austausch wichtig für die Reformbereitschaft: Der wachsende Einfluss soziologischer und anthropologischer Perspektiven steigerte das Interesse der Psychiater:innen, sich vermehrt mit den individuellen Geschichten und dem entsprechenden Krankheitserleben ihrer Patient:innen auseinanderzusetzen, so Söhner. Manche Akteur:innen empfanden diese Veränderung als bewusste Abwendung von biologischen Ansätzen, was zwar nicht unmittelbar zur Enquete führte, aber die Reformbereitschaft stärkte.

Söhners akribische Oral History gibt einen guten Überblick über die Entstehungs- und Entwicklungsgeschichte der Psychiatrie-Enquete und macht gleichzeitig das Erleben der Zeitzeug:innen greifbar. Die Ausarbeitung des Buches allerdings lässt Raum für Kritik. Ein genaueres Lektorat wäre sprachlich und stilistisch wünschenswert gewesen. Des Weiteren fällt an einer Stelle eine Ungenauigkeit in der Benennung psychopharmazeutischer Präparate auf, wo Wirkstoff- und Markennamen unglücklich vermischt werden.

Die Monographie *The Rise and Fall of the Age of Psychopharmacology* von Edward Shorter aus dem Jahr 2021 schließt an die Beiträge zur Psychopharmakakritik des Sammelbands von Borck und Lingelbach an. Während unter anderem Söhner darauf hinweist, dass Shorter vor allem für seine Erfolgserzählungen der Psychiatriegeschichte bekannt ist (Söhner 2019: 19), zeichnet das vorliegende Werk ein gegensätzliches Bild. Es stellt vielmehr eine Anklage gegen die Psychopharmakologie und die Psychiatrie dar, sich zu sehr an kommerziellen Interessen orientiert zu haben. Zwar sei die Möglichkeit, psychische Krankheiten (oder zumindest die Symptome) mit Medikamenten zu behandeln, ein „brillantes wissenschaftliches Ereignis“ (S. x),^1^ allerdings siegte dabei schlussendlich die Wirtschaft. Es kam zu einer „Pervertierung der Wissenschaft im Interesse der Wirtschaft“^2^ (S. x). Dies ist das zentrale Thema des Buches, das durch zahlreiche Fallbeispiele aus verschiedenen Kontexten erörtert wird. Die Monographie setzt bei der Vor- und Frühgeschichte der Psychopharmakologie an und beschreibt die Grundsteinlegung für die spätere Forschung zum Einfluss von Opioiden und anderen psychoaktiven Stoffen auf Gehirn und Verhalten im 19. Jahrhundert. Shorter legt dar, wie bereits im frühen 20. Jahrhundert Substanzen – beispielsweise Amphetamine und Kokain – in psychiatrischen Einrichtungen als Therapeutika genutzt wurden und sich im Lauf der Jahrzehnte das System aus den Diagnostiken Schizophrenie und Depression heraus entwickelte und festigte. Den Startpunkt der modernen Psychopharmakologie sieht Shorter in der Industrialisierung und zugleich Disziplinbildung dieses Wissenschaftszweigs – konkret in der Entwicklung von Chlorpromazin in den frühen 1950er Jahren, das international unter den Namen „Megaphen“, „Thorazine“, „Largactil“ und „Amplictil“ verkauft wurde. Schon für die 1950er Jahre weist Shorter auf die starken kommerziellen Interessen hin, die die Entwicklung und Vermarktung psychopharmazeutischer Produkte vorantrieben. Bezogen auf Ritalin schreibt er: „Es funktionierte für alles, wofür es vermarktet wurde.“^3^ (S. 55) Zwar bot die Arbeit mit diesen Substanzen der Wissenschaft die Möglichkeit, Wissen über die Zusammenhänge zwischen Stoff und Verhalten zu generieren, allerdings habe der Fokus zu sehr auf Industrie und Profit gelegen.

Während die frühen Entwicklungen insbesondere in Europa stattfanden, verlagerte sich das Kräfteverhältnis mit der Überarbeitung des *Diagnostic and Statistical Manual of Mental Disorders* (DSM) und des Erscheinens des DSM III 1983 in die USA. Neue Diagnosen wie schwere Depressionen („Major Depression“) und Antidepressiva wie Prozac wurden eingeführt. Die Legitimation für die Diagnosen lag zumeist in den Stoffen selbst, mit denen entsprechende Auffälligkeiten beeinflusst werden konnten. Shorter diskutiert im Anschluss an diese Entwicklungen detailliert, wie die Industrie durch sogenannte „Key Opinion Leaders“, den Aufbau von Studien, Marketing und wissenschaftliche Publikationen ihre Standpunkte und Interessen vertrat und verbreitete. Insgesamt versteht Shorter die Entwicklung der Psychopharmakologie als „wissenschaftliches Desaster“,^4^ das durch einen zu großen Einfluss der Industrie auf die Wissenschaft ausgelöst wurde.

Seine tiefen persönlichen Einblicke und Kontakte in das Forschungsfeld der Psychopharmakologie bieten einen guten, aber auch problematischen Ausgangspunkt für die Analyse. Die Beispiele sind fundiert und stringent präsentiert, allerdings erscheint der persönliche Bezug für eine wissenschaftliche Arbeit gelegentlich allzu stark. So führen umgangssprachliche Formulierungen an manchen Stellen dazu, das Argument eher wie eine persönliche Anklage klingen zu lassen. Ein weiterer Punkt, der in diesem Zusammenhang auffällt, ist die These, Effektivität hätte gegenüber Lukrativität das Nachsehen gehabt. Shorter begründet dies wenig überzeugend mit dem Argument, *One Flew Over the Cuckoo’s Nest* habe die wirkungsvolle Konvulsionstherapie desavouiert, in dem sie als gewalttätige Methode und als mögliches Instrument der Bestrafung inszeniert wurde. Auch wirksame Wirkstoffklassen seien aus kommerziellen Interessen heraus voreilig durch ineffektive, aber lukrative Behandlungsmittel wie Serotonin-Wiederaufnahmehemmer (SSRIs) ersetzt worden. Eins der berühmtesten Beispiele für einen solchen Stoff ist Prozac (Fluoxetin).

## Kritische Perspektiven auf die Deutungs- und Wirkmacht der Psychiatrie

In Kombination bieten die drei besprochenen Werke einen guten Einblick in relevante Fragestellungen der aktuellen Forschung und präsentieren darüber hinaus wegweisende Ansätze für die zukünftige Psychiatrie- und Psychopharmakologiegeschichte. Borck und Lingelbach, Shorter und Söhner weisen darauf hin, dass die zumeist gängige Geschichtsschreibung des Erfolgs in der Psychiatriegeschichte und der Geschichte von Psychopharmaka kritisch hinterfragt werden sollte. Dies kann sowohl durch eine tiefergehende Berücksichtigung marginalisierter Gruppen als auch durch eine Verbindung unterschiedlicher Ansätze der Geschichtswissenschaften geschehen. So können die vielschichtigen gesellschaftlichen, politischen, wirtschaftlichen und medizinischen Einflüsse multiperspektivisch aufgeschlüsselt werden.

Während sich sowohl Balz als auch Shorter schwerpunktmäßig mit der kritischen Entwicklung und Nutzung von Psychopharmaka in den 1960er bis 1990er Jahren auseinandersetzen, gibt es auch kritische Studien zu den Anfängen der modernen Psychopharmakologie in den 1950er Jahren. Viola Balz und Sandra Caponi beispielsweise untersuchen in ihren Arbeiten die Darstellung von Chlorpromazin, dem ersten Neuroleptikum, als Startpunkt der modernen Psychopharmakologie und als vermeintliche „psychopharmakologische Revolution“ (Caponi 2021) in der psychiatrischen Behandlung, die beide hinterfragen. Caponi argumentiert, dass trotz der vermeintlich revolutionären neuen Therapiemöglichkeiten durch Chlorpromazin der Schwerpunkt in den Anstalten nach wie vor auf der Ruhigstellung der Patient:innen gelegen habe. „Managing madness“ blieb Hauptziel, weswegen Caponi, ganz im Sinn des Sammelbandes von Borck und Lingelbach, dem Konzept eines revolutionären Wandels widerspricht (ebd.: 17). Auch Balz hinterfragt die Erfolgsgeschichte von Chlorpromazin und zieht dazu Patient:innenakten aus westdeutschen Psychiatrien heran. Sie arbeitet die unterschiedliche Wahrnehmung effektiver Wirkungen des Stoffs von Psychiater:innen und Patient:innen heraus. Während Betroffene das Neuroleptikum eher unvorteilhaft bewerteten, sahen Psychiater:innen darin ein wichtiges neues Hilfsmittel. Balz argumentiert, dass sich die Wirksamkeit des Medikaments nicht nur durch physiologische Effekte, sondern auch durch persönliches Erleben konstruierte. Durch die Analyse von Patient:innenakten kam sie zu der Ansicht, dass diese keineswegs die gängige Erfolgsgeschichte widerspiegeln (Balz 2010).

Die vorgestellten Studien zum Einfluss psychopharmazeutischer Stoffe in der zweiten Hälfte des 20. Jahrhunderts bieten einen wichtigen Anhaltspunkt für die Analyse der gesellschaftlichen und auch wirtschaftlichen Macht der Psychiatrie. Es zeigen sich nicht nur Entwicklungen und Strategien, mit denen die Psychiatrie sowohl im medizinischen Sektor als auch in der Gesellschaft an Einfluss gewann: Nach dem Anwachsen der Psychiatrien Ende des 19. Jahrhunderts, was mit der Auflösung bestehender sozialer Versorgungsnetzwerke in der Hochindustrialisierung zu tun hatte, spielte in der zweiten Hälfte des 20. Jahrhundert vor allem ihre Klinifizierung eine Rolle bei der Stabilisierung und Ausweitung ihrer sozialen Deutungs- und Wirkmacht hinsichtlich psychischer Normsetzung und -durchsetzung. Ihre Allianzbildung mit der Psychopharmakologie auf der klinischen Seite (gemeint sind vor allem klinische Arzneimittelforschungen) einerseits und die damit verbundene Ausweitung therapeutischer Möglichkeiten andererseits sicherte der Psychiatrie zugleich eine gesellschaftlich wie medizinisch bedeutende Rolle. Unterstützt durch wirtschaftliche Antriebsfaktoren entstand in diesem Zuge eine mächtige und einflussreiche Verbindung aus Psychiatrie und Industrie, die sowohl die medizinische als auch die gesellschaftliche Wahrnehmung nachhaltig prägte (Lenhard-Schramm et al. 2022; Shorter 2021).

Ein weiterer Punkt, der insbesondere die gesellschaftliche Wirkmacht der Psychiatrie zum Ausdruck bringt, ist der Umgang mit Betroffenen und vulnerablen Gruppen. In der medizin- und psychiatriehistorischen Forschung nehmen Forderungen nach mehr Gehör für jene Personen zu – seien es Kinder, Patient:innen oder Menschen mit Behinderungen. Von ihnen handeln die Erzählungen und Untersuchungen, allerdings kommen sie selten selbst zu Wort. Aktuelle Forschungsprojekte untersuchen verstärkt Quellen aus Betroffenensicht, wie die hier diskutierten Werke demonstrieren. Dabei ist die Herausforderung, derlei Quellen zu finden oder entsprechend zu deuten, nicht trivial, da sie nicht immer aus erster Hand verfasst wurden (França 2014: 106). Studien aus Sicht vulnerabler Gruppen vorzunehmen heißt nicht nur, die gängige Geschichtsschreibung zu hinterfragen, sondern auch, neue Perspektiven und Denkweisen zu ermöglichen. Borck und Lingelbach thematisieren die enge Verbindung zwischen psychisch kranken und Menschen mit Behinderung. Diese Nähe im Sinne gesellschaftlicher Hürden und Ausgrenzungsbewegungen wird auch bei Tiago Henrique França deutlich. Er diskutiert am Beispiel von Menschen mit Behinderungen gesellschaftliche Normvorstellungen von Körpern, die Interpretation von Behinderungen als unweigerliches Hindernis und die damit einhergehenden gesellschaftlichen Diskriminierungsprozesse (ebd).

Globale Prozesse können lokal unterschiedliche Ausprägungen und Auswirkungen haben und ebenso können lokale Eigenheiten auf globale Prozesse Einfluss nehmen. Dies gilt auch für psychiatrisch-wissenschaftliche Entwicklungen (Stuchtey & Wiegeshoff 2018; Antić 2022). Wie Borck und Lingelbach sowie Söhner zeigen, führten die erstarkende Kritik und die Sensibilisierung für etwaige Missstände ab den 1960er Jahren zu umfassenden, wenn auch nicht unmittelbar erfolgreichen Bewegungen und Veränderungen in der psychiatrischen Landschaft. Dies ist aber eine primär westliche Perspektive, die dem historiografischen Desiderat einer zunehmenden Multiperspektivität der Geschichten der Psychiatrie auf einem globalen Maßstab wenig gerecht wird. In gewisser Weise kann ein globaler und postkolonialer Blick auch die wiederum westliche Erzählung des schwindenden Einflusses psychiatrischer Normsetzungen und der Enthospitalisierung konturieren. Während aus westlicher Sicht die gesellschaftliche und politische Wirkungsmacht der Psychiatrie anhand der Diskrepanz zwischen zunehmender Psychiatriekritik bei gleichzeitiger Ausweitung medikamentöser Behandlungsmethoden und Diagnostiken, auf die Shorter exzellent verweist, zu diskutieren sind, ergibt sich im Fall Brasilien eine andere Diskussionsgrundlage (Zorzanelli et al. 2014). Eine historische Analyse der Militärdiktatur Brasiliens zwischen 1964 und 1985 zeigt, wie psychiatrische Institutionen, Diagnostiken und Methoden von politischen Regimes eingebunden und genutzt werden können. Nicht nur die mögliche Instrumentalisierung therapeutischer Methoden wie der Elektrokonvulsiven Therapie (ECT) zur Folter (Guedes 2019: 69, 75, 84, 126) und die mögliche Anwesenheit von Psychiater:innen oder Psychoanalytiker:innen in Foltergefängnissen (Coimbra 2004: 49; Moreira et al. 2014) verdeutlicht die enge Verbindung von Psychiatrie, Politik und Gesellschaft und die damit einhergehende gesellschaftliche und politische Macht der Psychiatrie. Auch die Pathologisierung und Medikalisierung des „Subversiven“ – politischer Gegner:innen, die insbesondere linkspolitische und kommunistische Ansichten vertraten (Guedes 2019: 58) –, unterstreicht diese mächtige Verbindung. Neben ihrer politischen Verfolgung kam es zu einer Assoziation mit dem „Drogado“ – dem Drogenabhängigen (Coimbra 2004). Drogenabhängigkeit als Krankheit wiederum fiel in den Zuständigkeitsbereich der Psychiatrie. Auf diese Weise wurden psychiatrische und medizinische gezielt mit politischen Themen in Beziehung gesetzt und somit der Medikalisierung potenzieller politischer Gegner:innen Raum gegeben. Die selbst während der Diktatur verfolgte Psychologin und Gründerin des Vereins *Tortura Nunca Mais* Cecília Maria Bouças Coimbra schildert, wie die Konzepte des Subversiven und des Drogenabhängigen mit Attributen wie „krank“, „unangepasst“ und „unstrukturiert“ verbunden und damit in den Einzugsbereich der Psychiatrie sowie der Psychoanalyse eingegliedert wurden (Coimbra 2004: 45). Regimekritische Personen konnten als behandlungsbedürftig eingestuft und in entsprechende Einrichtungen verbracht werden (ebd.: 50; Faria 2015: 224–225; Guedes 2019: 53). Während in der westlichen Psychiatriegeschichte die 1970er und 1980er Jahre insbesondere als psychiatriekritische, reformorientierte Jahrzehnte diskutiert werden, verzögerte sich diese Entwicklung in Brasilien. Zwar erreichten kritische Perspektiven auch Brasilien, jedoch wurden diese während der Diktatur verstärkt unterdrückt. Erst Ende der 1970er und vor allem nach der Diktatur in den späten 1980er Jahren wurden Maßnahmen zielgerichtet diskutiert und 2001 gesetzlich umgesetzt (Ministério da Saúde 2021).

## Fazit

Die Psychiatrie besitzt nicht nur eine medizinische, sondern auch eine gesellschaftliche und politische Wirkmacht, die sich lokal und zeitlich unterschiedlich äußert. Dies ist sowohl ein historisches als auch ein aktuelles Phänomen, dem sich die Psychiatriegeschichte zurecht intensiv widmet. Sowohl Medizin- und Psychiatriegeschichte als auch Gesellschafts‑, Global- und Wirtschaftsgeschichte haben einiges zu diesem Themengebiet beizutragen. Cornelius Borck, Gabriele Lingelbach, Edward Shorter, Felicitas Söhner und andere Wissenschaftler:innen verdeutlichen, wie durch eine interdisziplinäre Ausrichtung, durch Einbindung der Oral History und durch die Fokussierung auf die Stimmen und das Erleben Betroffener und Beteiligter die vorherrschende Geschichtsschreibung sowie die zahlreichen Erfolgsgeschichten in Psychiatrie und Psychopharmakologie kritisch hinterfragt und neue Forschungsperspektiven geöffnet werden können. Auf diese Weise kann das komplexe und vielschichtige Thema Psychiatrie in seinen zahlreichen Facetten umfangreich untersucht, die einzelnen Mosaike miteinander in Beziehung gesetzt und zu einem Gesamtbild zusammengefügt werden. Allerdings ist die globale Ungleichzeitigkeit der Medikalisierung und Demedikalisierung nach wie vor ein Forschungsdesiderat, das größere Aufmerksamkeit geschichtswissenschaftlicher Disziplinen verdient. Die diskutierten Arbeiten und der Blick Richtung Brasilien zeigen, dass nicht nur Medizin- und Psychiatriegeschichte angesprochen werden, sondern dass es sich um ein vielschichtiges und komplexes Forschungsfeld handelt, das zu interdisziplinärer Arbeit auffordert.

## Footnotes

1 Im Original: „brilliant scientific event“.

2 Im Original: „perversion of science in the interest of commerce“.

3 Im Original: „It worked for whatever it was marketed for.“

4 Im Original: „science disaster“. Es handelt sich um die Überschrift von Teil 3.
